# A Metasynthesis on Perceptions of Sexism Among Video Game Players and the Video Game Industry

**DOI:** 10.3390/bs16030319

**Published:** 2026-02-26

**Authors:** Javier Denia Mondéjar, Vanesa Pérez-Martínez, Carmen Vives-Cases

**Affiliations:** 1 Public Health Research Group, Department of Community Nursing, Preventive Medicine and Public Health, History of Science, University of Alicante, Carr. de San Vicente del Raspeig, s/n, 03690 Alicante, Spain; jdm41@alu.ua.es (J.D.M.); carmen.vives@ua.es (C.V.-C.); 2 CIBER of Epidemiology and Public Health (CIBERESP), 28029 Madrid, Spain

**Keywords:** video games, sexism, metasynthesis, gamer community

## Abstract

Objective: This metasynthesis analyzes the current evidence on the perceptions of sexism among video game players (men and women) and the video game industry. Methods: The databases Scopus and ProQuest were used to select 15 qualitative studies in a final analysis. The analysis used the reciprocal translation technique to analyze and interpret the data. Results: Three primary themes emerged: normalized violence in gamer environments (*n* = 13), strategies to confront sexism/gender harassment (*n* = 10), and internal ambivalence and tension (*n* = 10). The results show that female videogame players experience daily hostility due to the fact of being women in an environment of male dominance. This leads to recurring confrontations, which can result in female video game players being questioned in terms of identity and behavior by both themselves and the community. Conclusions: This metasynthesis suggests that sexism in online video game environments is not an isolated phenomenon and directly affects the experience of female video game players. Furthermore, it highlights the importance of further exploring the perceptions and experiences of different groups within gamer communities, in order to make progress towards a more egalitarian and diverse culture.

## 1. Introduction

In the last decade, the video game industry has been consolidated as a global entertainment and leisure sector, both economically and in terms of players. According to the report published by Video Games Europe (2024), in 2023 the European video game market was valued at 25.7 billion euros, with 124.4 million players. Of these, nearly half are women (43.5%). It is estimated that by 2026 the video game market will have generated around 184 billion dollars, with 3.38 billion players around the world (Snegiev, 2023). Furthermore, Spain is an important part of the sector (AEVI, 2023), both economically and socially, with 2.239 billion euros in sales in 2023 and 20.05 million players. Of this total, 51% are men and 49% are women. As shown, there is a near gender parity in the aggregated data of people who play video games. However, this apparent parity masks a significant demographic divide when age and gender are analyzed concurrently. Data from the previous report (Video Games Europe, 2024) indicates that while video games are a near-universal leisure activity for youth, with 83% of those aged 11–14 being active players, the female representation within the 6–17 age bracket stands at a 26%. This gap suggests that structural and social barriers may be discouraging girls from entering the gaming ecosystem at an early age. Interestingly, female participation grows significantly with age, reaching 32% among 18–34-year-olds and 42% in the 35–64 age group. This latter segment is particularly crucial, as the 45–64 age bracket constitutes the single largest demographic of the market, representing 23% of all players. These data suggest that the gaming ecosystem is not a neutral environment, but rather a socially constructed space where participation is heavily influenced by external cultural factors.

Consequently, this digital realm has become a new way of relating to others. However, this leisure space is not free from problems: the problems that appear in society are transferred to digital society. Patriarchy moves into of video games context, giving rise to a realm dominated by hegemonic masculinities (Díaz-Fernández & García-Mingo, 2024). As documented in the previous paragraph, nearly half of the player population are women, and despite this, they continue to have difficulties entering, and even remaining in, the sector. Therefore, it is important to understand how sexism plays a key role in the perpetuation of patriarchy within gamer culture. It is necessary to understand not only how it affects women and the strategies they use to confront it, but also why there are men who continue to perpetuate it, how it affects men who do not, and what effective strategies there might be for eradicating it.

### 1.1. Sexism: Glick and Fiske’s (1996) Theory of Ambivalent Sexism

From a theoretical perspective, Glick and Fiske (1996) defined sexism as a set of attitudes, prejudices and beliefs based on gender that perpetuate inequality between women and men. The authors argue that sexism not only has a hostile or “negative” component, as had been theorized previously, but can also be formed by benevolent or “positive” attitudes or beliefs. Despite these attitudes being apparently positive, they remain harmful, as they perpetuate gender inequality. This is what they called Ambivalent Sexism, which is composed of two dimensions.

On one hand, hostile sexism refers to those attitudes, beliefs or stereotypes that are openly negative toward one sex, mainly toward women. For example, beliefs such as women being inferior, or misogynistic attitudes toward them, are the main component of hostile sexism. This form of sexism is primarily motivated by the growing participation of women in masculinized society and in the fight for their rights, including men’s belief that women want to control them (Glick & Fiske, 2001). One form of hostile sexism can be found in Incel groups (acronym of “*involuntary celibate*”), defined as young men with misogynistic and often violent tendencies, through both traditional and internet contexts (Byerly, 2020). In contrast, benevolent sexism encompasses those beliefs or attitudes toward women that appear to be positive but are not. For example, thinking that women should be protected by men since they are “weaker”, the belief in women’s inherent capacity to care for others, or the emphasis on female beauty. This sexism perpetuates gender stereotypes in a “positive” way that maintains patriarchy through a benevolent logic (Barreto & Ellemers, 2005). One form of benevolent sexism can be seen in the *Tradwife* movement. In maintaining and perpetuating the patriarchal and sexist system, these two dimensions act in a joint way (Glick & Fiske, 2001).

### 1.2. Manifestations and Prevalence of Sexism in Online Video Games

In gaming environments, sexism can manifest in different ways, ranging from explicit harassment behaviors to more subtle attitudes of exclusion or the hypersexualization of female characters. Explicit sexism refers to overt, conscious, and often aggressive expressions of gender-based violence. One of the most common expressions is verbal and written harassment of a sexist nature, or what is known as *gender trash talking* (Vergel et al., 2023). This expression of sexism involves a series of attitudes toward women such as casual public insults, hostile comments, verbal abuse or dismissive interactions based on their performance in games, but which are motivated by being women or their perceived gender. In these digital spaces, gendered slurs and epithets act as everyday forms of containment aimed at policing behavior that is deemed insufficiently feminine or masculine. These slurs are not merely insults; they are derogatory terms motivated by hostility that actively attempt to degrade the target’s confidence, authority, and power. With-in game chats, women face a wide range of attacks that target their intelligence with terms like “bimbo” or “stupid”, attack their sanity with words like “hysterical” or “psycho”, insult their physical appearance, or reduce them to domestic service tasks, such as calling them a “dishwasher” (Miller-Idriss, 2025).

Sexual harassment is another expression of sexism that women face. Sexual harassment can take the form of attitudes such as sexist jokes or comments and insults, comments about the player’s appearance or jokes about rape and abuse (W. Y. Tang et al., 2019). In fact, graphic rape and death threats have become a standard discursive move used to express disapproval of women online. The primary aim of this extreme, sexually explicit rhetoric is not just to challenge women’s presence, but to scare and silence their voice entirely (Jane, 2014). Furthermore, it is important to recognize that this harassment is often layered. This is what Crenshaw (1991) calls intersectionality. Intersectionality framework illustrates how various forms of systemic bias overlap. It highlights the unique challenges faced by individuals whose identities span multiple marginalized groups (such as race, ethnicity and sexual orientation) resulting in specific experiences of discrimination. The mechanism of explicit sexism is often rooted in a social dominance way where individuals (typically men) perceive the entry of women into a traditionally masculinized domain as a threat to their status. Research suggest that violence is often a reaction to perceived threats against patriarchal structures, particularly when men feel that their systemic dominance is being questioned (Hunter & Jouenne, 2021).

Additionally, implicit sexism is more insidious, operating through subconscious biases and seemingly positive attitudes that nonetheless perpetuate inequality. Unlike hostile sexism, benevolent sexism operates through seemingly positive and well-intentioned attitudes. However, these subconscious biases are deeply patronizing, as they inherently portray women as fragile individuals who require support and protection. This framework reveals how inequality is perpetuated not just through open hostility, but through restrictive, paternalistic attitudes that limit female independence (Warr, 2022). This implicit sexism often manifests by placing female characters into culturally safe and acceptable roles rather than allowing them to by dynamic and agentic. They tend to be passive rather than active, rarely making their own choices or clearly voicing their own ideas (Bègue et al., 2017; Perreault et al., 2018). The perpetuation of these subtly sexist topics has a measurable, insidious impact on those who consume video games. Previous research suggests that exposure to video games perceived as sexist is correlated with higher rates of benevolent sexism among men that plays those videogames (Stermer & Burkley, 2015). As mentioned previously, stereotypes do not exist only in real life, but are also transferred to the digital realm, in addition to being created or transformed. One of the most common stereotypes in the gamer community is the belief that women are less skilled at playing or that they cannot play competitive video games (Kelly et al., 2022). This stereotype can manifest in behaviors such as ignoring women’s contributions in cooperative matches, not trusting them for key roles within the game or being surprised when a woman excels, attributing her success to external factors instead of her skill (D. Tang et al., 2025).

Regarding the prevalence of these sexist manifestations, evidence suggests that sexism is a systemic problem and not limited to isolated incidents. A report conducted by McBean (2020) reveals that more than half of respondents who play video games (58% of women and 62% of men) have experienced some type of abuse when playing online. However, the same study reveals that women are more vulnerable to experiencing inappropriate sexual behaviors and sexist attitudes on behalf of men. For example, 27% of women have been excluded from participating in a game solely because of their gender, compared to 14% of men. Additionally, 28% of women have experienced some type of sexual harassment by men.

### 1.3. Consequences of Sexism in Online Video Games

Sexist behaviors in video games not only generate uncomfortable and unpleasant situations but have consequences at a psychological and social level. Various studies have explored the impact that the sexist environment has on female players, revealing negative effects on their wellbeing and behavior. At a psychological level, there is evidence that facing harassment and contempt constantly can lead to stress, anxiety, low self-esteem, etc. A study conducted by McLean and Griffiths (2019) suggested that many women come to experience anxiety and feelings of loneliness due to this hostility and the lack of support in matches. Furthermore, insecurity is a constant feeling for women, as they have to make more effort to demonstrate their skill or their ability to play video games. Sometimes they even begin to believe that “they are not good at it.” These psychological consequences affect how women interact with others during their gaming sessions, which leads to consequences at an interactional and behavioral level.

At the social and behavioral levels, women seek strategies to be able to confront the violence they experience. In a scoping review, Vergel et al. (2023) analyzed the consequences of cybersexism and how it affects female *gamers*. They found that women use a series of strategies to either confront sexism or distance themselves from video games. The most common is called *gender masking* or gender concealment. These practices are described as preventive risk-management strategies, where women use masculine pseudonyms or avatars to avoid potential sexist interactions. Another strategy used is avoiding online gaming. Many women decide not to play video games online or to play only solo, due to their perception of the potential risks that playing online games has for them. Although uncommon, some decide to directly confront the sexism they experience by responding to the aggressor. When women directly confront their aggressor, they are often singled out and excluded, with the aggressor even receiving support. In this sense, evidence suggests that there is a strong correlation between the underrepresentation of women in *eSports* and the prevalence of gender-based harassment they face (Ruvalcaba et al., 2018). Toxicity even becomes normalized, as it is accepted as part of *gamer* culture (Türkay et al., 2020). This normalization of violence stems from a masculinized culture, where violence is used as a competitive resource. In this way, sexism reproduces gender stereotypes whereby men have to be strong and brave, and women have to be weak, submissive and obedient. This makes many women feel unfit to enter or remain in the world of video games, together with other factors such as lack of social support or hostile and violent situations toward them joining in.

### 1.4. Relevance of the Study

Despite the severity and complexity of the problem, there is a lack of academic research on sexism in video games. Specifically, the lack of qualitative information hinders the understanding and subjective comprehension of the players’ experiences of sexism. A large part of the studies that have been conducted to date have approached the problem through surveys or quantitative methodologies, to analyze the prevalence of harassment, the behaviors women have witnessed or the strategies they use to confront it, especially in regard to quantitative reviews (Vergel et al., 2023; García-Naveira et al., 2022; Yunhao et al., 2025). Thus, there is a gap in qualitative reviews about how and why sexism is experienced in the day-to-day environment of the *gamer* community. Despite the rapid growth of the female population in the video game sector, there is still a significant gap between the diverse *gamer* reality and the persistent masculinity of *gamer* culture, which hinders the full participation and recognition of women or other non-heteronormative realities (Scidone et al., 2024). On the other hand, most research that attempts to analyze sexism in video games focuses solely on women (Blackburn & Scharrer, 2018). Including men and masculinity in this type of research is fundamental to an in depth understanding of why some men do not express sexist behaviors or how it affects them when they are victims of these behaviors themselves, whether on behalf of other men or by women. Additionally, there is little information about the perceptions of the video game industry, which is a key element in eradicating sexist behaviors, as the industry has the capacity to moderate and sanction the behavior of players. 

The main objective is to analyze the existing evidence in the literature on the perceptions of sexism among female players, male players and the video game industry. Likewise, the specific objectives of the present study are as follows: (1) to analyze the experiences of players and the industry regarding sexism in video games; (2) to understand in-depth the perceptions of video game players and how they confront or are affected by sexism; and (3) to reflect on the social and cultural implications of sexism in the world of video games and thus propose solutions or future lines of research. To achieve these objectives, three research questions have been formulated:

1. What sexist violence do players experience in the realm of online video games?
2. What strategies do players use to confront sexism in online gaming environments?
3. What are the consequences for players who experience sexist violence within online video games?

## 2. Methodology

### 2.1. Qualitative Metasynthesis

Metasynthesis is a type of review study that integrates results from qualitative research with the objective of formulating new data in relation to the topic addressed. It does not attempt to integrate or group results from other qualitative research, but rather to make an interpretative analysis of the results obtained by other qualitative studies (Jensen & Allen, 1996). Therefore, the objective of conducting a metasynthesis is not to summarize the results, but to cover a topic in more depth, through the interpretation of different results (Suzuki & Yuan, 2021).

### 2.2. Sources of Information and Search Strategy

The search was conducted in the Scopus and Proquest databases during January and February of 2025. To undertake a quality review, the search and methodology were carried out based on the PRISMA recommendations (Page et al., 2021) and the JARS-Qual standard for qualitative reviews (American Psychological Association, 2024). The search equation used was the following: [(sexism OR cybersexism) AND (gaming OR videogame* OR game*) AND (online OR internet OR “in line”) AND (qualitative) NOT (sport*)]. There was no restriction on publication date. Additionally, the search was adapted depending on whether it was used for Proquest or Scopus.

### 2.3. Eligibility Criteria

The inclusion criteria included: (1) articles that refer to sexism in online video games; (2) qualitative studies or those with mixed methodologies with women and/or men or those that use other types of qualitative analysis (such as text messages, online chats, forums, discussion threads, etc.); and (3) articles published in English or Spanish. In addition to the previously mentioned inclusion criteria, the following exclusion criteria were defined: (1) articles that do not specifically address sexism in the context of online video games; (2) studies that do not use qualitative or mixed methodologies.

### 2.4. Study Selection

**Figure 1:**
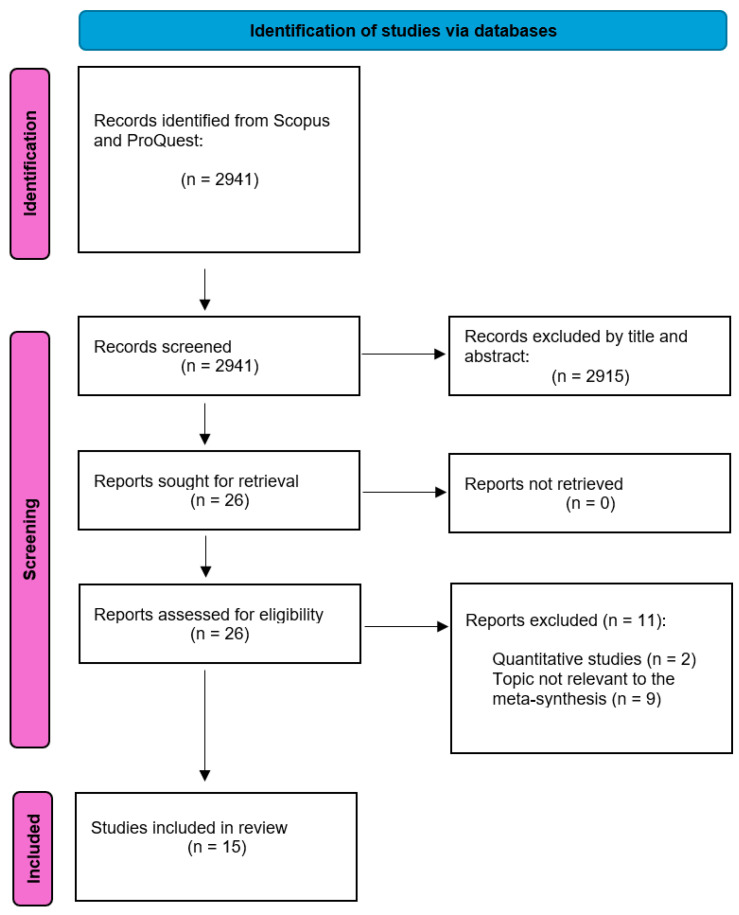
PRISMA flow diagram (Page et al., 2021).

A total of 2941 articles were extracted from the two databases mentioned. Two members of the research team independently and critically examined the abstract and title of each article and discarded those studies that did not meet the inclusion criteria. In this phase, 26 articles were selected and then included in the Zotero Program for better organization. Subsequently, a member of the team carried out a complete critical reading of the selected studies. Thus, 11 of the 26 articles were excluded due to: (1) quantitative study (*n* = 2); and (2) does not respond to the objective of the metasynthesis (*n* = 9). A total of 15 articles were compiled (Table 1). Figure 1 shows the flow diagram of the article selection process.

### 2.5. Quality Analysis

All articles selected for the metasynthesis were analyzed with the quality assessment instrument, the *Mixed Methods Appraisal Tool* (MMAT; Hong et al., 2018). This instrument provides response categories for each methodological criterion (“yes,” “no” or “I don’t know”). To evaluate qualitative studies, this instrument uses questions related to the adequacy of the methodology to answer the research question, adequacy of data collection methods, results derived from data analysis, and interpretation of results grounded in the data and coherence between these elements.

### 2.6. Data Extraction and Analysis

For the extraction and analysis of data, the concepts proposed by Noblit and Hare (1988) were followed. Specifically, the reciprocal translation technique was used. This technique consists of understanding the results of one study in terms of the findings of other studies with the objective of developing consistent syntheses among the included research. Furthermore, this technique has been used in most metasyntheses (Melendez-Torres et al., 2015).

The process was as follows: first, the main themes that emerged from each study were analyzed through the researchers’ findings and the participants’ quotes; then, the relationship between the emerging themes from each study was analyzed to note a first interaction into more general themes, and; finally, a final interaction was conducted to extract the results into final themes. This process was adapted from Walji et al. (2014). According to Paterson (2001), it is important to ensure the transparency of the process. Thus, the process was carried out in Microsoft Excel, where the main themes were addressed manually by the principal investigator. Table 2 presents the interaction of the themes.

## 3. Results

Table 3 shows the quality analysis of the selected articles, indicating a solid design, analytical coherence and an adequate use of qualitative techniques to respond to the research objective or question. Through the use of the *reciprocal translation* technique and a complete reading of the studies, three themes were identified: (1) Normalized violence in gamer environments; (2) Strategies for coping with sexism/gender-based harassment and; (3) Ambivalences and internal tensions.

### 3.1. Question 1: Normalized Violence in Gamer Environments

This heading groups those studies where participants experienced sexist violence or have witnessed it. Most of the selected studies address this theme, representing 87% of the total (*n* = 13). All of them analyze, globally or specifically, the violence that is committed in online video game communities and that is directed specifically toward women. Additionally, one study analyzes the perceptions that men have about sexism and the violence that exists in *gamer* culture (Ortiz, 2018).

(a) Harassment and gender trash talking

Several studies highlight how the most normalized violence within online *gamer* spaces is made up of sexist comments, verbal discrimination or misogynistic insults. This is called trash-talking, specifically gender trash-talking, since it is motivated by gender. (Crothers et al., 2024; Deng, 2024; Giolla, 2018; S. J. Kim & Kim, 2022; J. Kim & Ortiz, 2024; Moura et al., 2024). Deng (2024) shows how participants refer to three types of interaction with men in online matches, the first refers to insults or misogynistic labels such as “whore” or “bitch”, followed by sexual objectification and sexual violence toward women. As one participant notes: “It’s gamer culture… these insults are considered a part of the sport, even at the cost of someone’s wellbeing… we all learn to talk like this” (Emiliano, in Ortiz, 2018, man, p. 8).

Furthermore, Giolla (2018) shows how sexism and gender-based harassment in video games are often normalized within player interactions, to the point of being confused with “acceptable” behaviors characteristic of gamer culture. Humor serves to mask sexist attitudes, blurring the line between jokes and harassment, which trivializes these forms of violence. The author analyzes messages such as “if you choose to expose yourself to that environment, you should learn to hit back instead of crying on the internet” (Anonymous, p. 127) or “toughen up, if you’re offended by people you choose to play with, it’s partly your fault” (Anonymous, p. 127).

This culture of symbolic violence is also reproduced in competitive environments such as eSports. Moura et al. (2024) document the way in which many female players experience persistent forms of harassment and discrimination. For example, one participant spoke about how, upon reaching a high rank, she was automatically accused of having paid another player, (elojob) because it was not accepted that a woman could excel on her own merit (p. 21). Additionally, other interviewees describe feelings of discomfort related to sexist insults and constant romantic or sexual insinuations, which question their legitimacy as players and reinforce gender stereotypes.

These forms of violence also extend to other spaces linked to the industry, such as education, *gamer* events and the work environment. In the academic sphere, women report experiences of public ridicule and questioning of their presence in classrooms: “The professor chose to humiliate the only girl in the class on the first day. Me” (Woman, in Ochsner, 2017, p. 11). At conferences and events, many point out that it is assumed that they are companions or models, their contributions are ignored and they suffer physical or verbal harassment: “everyone assumes you’re someone’s girlfriend” (Woman, in Ochsner, 2017, p. 11). These reports show how sexism is deeply rooted in the different levels of *gamer* and professional culture.

Finally, Ortiz (2018) analyzes male perceptions of toxicity. Several men justify the use of violent language as part of competitive *gamer* culture, without recognizing its sexist insinuations. As one participant notes, these expressions “fit into the atmosphere of wanting to dominate” (Leonard, Man, p. 9) shedding light on a lack of awareness about the structural component of these forms of violence.

(b) Exclusion as the norm

Linked to trash talk and harassment, experiences of exclusion are also frequent (Choe et al., 2020; Crothers et al., 2024; Fousek & Švelch, 2024; Harvey, 2021; Moura et al., 2024; Naidoo et al., 2020). In eSports, women who aspire to participate in competitive teams are pushed aside by their male teammates. Crothers et al. (2024) highlights these experiences by interviewing women who participate or have actively participated in a professional team: “I tried out for a team a couple of years ago and everything seemed to be going well, but in the end they told me: yeah, the truth is we don’t want a girl to play on our team” (P6. p. 5). The same exclusion experiences appear in video game journalism. For a woman to be able to work professionally in video game journalism, she has to play more and know more than a man (Fousek & Švelch, 2024). Martina recounts how she was systematically ignored in professional meetings: “I had the strong feeling that I was just decoration. When I asked a question, they would answer my male colleague” (Woman, p. 461).

As a result of this exclusion, Choe et al. (2020) show how women feel less valid as gamers, which reinforces a cycle where they trust less in their abilities when playing with men, trying to focus only on the support role. Along the same lines, Naidoo et al. (2020), based on a local forum of an online video game community, identify 4 specific forms of exclusion present in these environments: (1) exclusion due to harassment; (2) exclusion due to stereotypes; (3) exclusion due to masculine hypercompetitiveness, and (4) exclusion from employment networks in the video game industry.

(c) Sexualization of women

The sexualization of women as a form of symbolic violence is another manifestation of sexism in gamer culture. The results show that this manifests on two levels: on one hand, the aesthetic hypersexualization of female characters within the games themselves; on the other, the application of double standards toward female players or video game industry professionals, who are subjected to constant evaluations based on their appearance or way of dressing, both in online spaces and in face-to-face interactions.

Participants in a study by Choe et al. (2020) show how one of the most visible and persistent forms of this symbolic violence is the sexualized representation of female avatars in video games. As they explained, female characters usually present scanty clothing, with stylized and exaggerated bodies, whose function does not respond so much to the logic of the game but rather to satisfy a sexualized male gaze. Regarding this sexualization, men tend to have two discourses: For example, Jihu (Man, in Choe et al., 2020, p. 47) expresses the following:

Female characters always seem sexual. We can see under the skirts of female characters; the more they expose their bodies, the greater their defense power… male characters don’t go around in underwear. Male characters are dressed in uniforms while women are naked… They appear to be sexual objects.

However, some mention that male characters are also sexualized according to women’s tastes, so it is normal for female characters to also be sexualized according to men’s tastes:

Those are things that women, stereotypically, find sexy (except for the excessive muscles thing, but for some reason men often want to look like that, so the same argument applies), just as men, stereotypically find slim and curvy women sexy… (Dirk, Man, in Naidoo et al., 2020, p. 590).

Beyond the design of female characters, some studies show how female players themselves suffer constant surveillance regarding their appearance and way of dressing, which also contributes to reinforcing their subordinate position within *gamer* communities. Ochsner (2017) shows that women are evaluated by criteria that are very rarely applied to their male colleagues: “I have to spend twice as much time choosing what to wear for *gamer* events as for a date” (Woman, p. 8). This type of pressure responds to a logic where the female presence in the *gamer* space is considered as a complement to men. In this research some of the interviewees stated that they deliberately modify their appearance to reduce sexualization or to avoid being perceived as objects of desire: “I changed things about how I dress, even my glasses, to be seen as an equal and not as a piece of meat” (Woman, p. 8).

### 3.2. Question 2: Strategies for Coping with Sexism/Gender-Based Harassment

The hostile situations that female players experience have led many to develop specific coping strategies with the objective of protecting themselves emotionally and socially, as well as preserving their participation in spaces that, on many occasions, exclude them. Across studies, these strategies range from individual risk-management to collective forms of mutual support. Therefore, this section refers to those articles that discuss how women confront sexism. Specifically, 67% of the analyzed articles (*n* = 10) address this theme (Choe et al., 2020; Cote, 2015; Crothers et al., 2024; Deng, 2024; Fousek & Švelch, 2024; Harvey, 2021; J. Kim & Ortiz, 2024, Kuss et al., 2022; McLean & Griffiths, 2019; Moura et al., 2024).

(a) Avoidance and risk-management strategies

One of the most common ways in which female players confront sexism in video game environments is through passive avoidance strategies. These consist of minimizing their visibility or limiting their interaction with the gamer community as a mechanism of self-protection and prevention. The most frequently observed in most studies is gender concealment or *gender masking* (Cote, 2015; Crothers et al., 2024; Deng, 2024; Fousek & Švelch, 2024; Kuss et al., 2022, McLean & Griffiths, 2019). This strategy consists of hiding women’s identity, by avoiding using voice chat or choosing neutral usernames so as not be recognized as women or abandoning their “feminine tone”: “My username doesn’t make it clear that I’m a girl, and there are times I don’t use the microphone, so people don’t really know I’m a girl” (Arya, cited in Cote, 2015, p. 9).

Participants in Deng’s (2024) study emphasize the use of this strategy for prevention, and not so much as a reaction to sexism. Participants show a clear idea of *gamer* culture: constantly confronting sexism is useless and exhausting, so they opt for preventive strategies. This is reinforced in the analysis by McLean and Griffiths (2019) where women admit to hiding their identity with the use of neutral avatars and pseudonyms to avoid negative behaviors toward them: “I have almost completely hidden my gender for the last 10 years in online games to be able to enjoy my hobby” (post number 190, Woman, p. 983).

Another passive strategy that women use to prevent sexism is abandoning online games (Cote, 2015; Deng, 2024; S. J. Kim & Kim, 2022). Some women prefer solo games or games with friends in controlled spaces, perceiving them as safer:

I don’t have an online account. I used to play a bit with my brother’s account, but then the unpleasant players showed up. I also used to play with my boyfriend’s account, and then I encountered unpleasant players. So yeah, I don’t like the culture that surrounds online multiplayer games

(Feather, Woman, in Cote, 2015, p. 8)

Finally, S. J. Kim and Kim (2022) show how many women who aspire to compete in online environments start out motivated and enjoy the game, but end up withdrawing due to a hostile environment full of prejudice, not for lack of skill.

(b) Active confrontation responses

While many female players opt for avoidance and risk-management strategies, others decide to confront sexism actively. These responses seek to protect individual integrity and also confront the structural discrimination that surrounds *gamer* communities. Seven studies address these responses, from demonstrating skills and ability, to fighting against aggressors by showing aggressive or strong personalities (Choe et al., 2020; Cote, 2015; Crothers et al., 2024; Fousek & Švelch, 2024; Harvey, 2021; J. Kim & Ortiz, 2024).

Many women emphasize their skills more noticeably than men, due to the pressure to prove their legitimacy as players. Some women affirm that when they are harassed or violated with sexist comments, they defend themselves by stating that they’re envied because they are better or that they have been playing the game for a long time: “I’ve been playing since the game came out (vanilla)” (Alissa, Woman, in Cote, 2015, p. 11). Others decide not to speak, but over-emphasize their skills so that players really see them as players. This represents extra pressure for female players, potentially affecting their mental health and consequently giving rise to other avoidance strategies such as abandonment.

In games where roles are very marked (support, damage or tank) women are relegated to support roles where they can only play female characters. Due to this, many women must make the extra effort to show their male teammates that they can also play other characters:

I play support, so it’s obvious that that already puts me in that group of people “Oh, she only plays Mercy (healer in the video game Overwatch)” but I hate playing Mercy. I prefer to play characters like Lucio or Baptiste (male support characters) you know, play normal support characters and that took a lot of persuasion, like doing tryouts and stuff, to tell people that, look, I really can play these support heroes

(P6, Woman, in Crothers et al., 2024, p. 6).

Some women opt to align themselves with the “competitive male *gamer*” to gain acceptance, either by imitating masculine *gamer* aesthetics or stating that they feel “more like one of the guys” (Harvey, 2021). This strategy, however, can reinforce hegemonic masculinity. In this sense, it is less common to use direct confrontation through aggressive personalities. Cote (2015) shows how there are women who claim to assume aggressive or sarcastic personality traits to be able to defend themselves effectively, even gaining respect from their aggressors:

I never acted like they thought I would, so I didn’t cry or complain saying: Oh my god, how cruel you are! I was a jerk to them… and that, somehow, has earned me a lot of respect, because they know I don’t let myself get walked all over

(Elizabeth, Woman, p. 12).

Finally, the only study that shows differences in the confrontation strategies used by men and women (Choe et al., 2020) shows that men tend to act more impulsively when attacked with toxic or sexist language. In this way, they come to use the same language or the same insults: “If the opponent’s tone is offensive, I get angry and fight back…” (Jaheon, Man, p. 49).

(c) Social Support and solidarity networks

Avoidance and risk-management strategies and active confrontation responses respond to the need for female players to protect themselves and resist violence and sexism in *gamer* environments. However, five of the studies mentioned in this section agree in pointing out that the most effective and sustained resource over time for confronting these experiences is social support (Harvey, 2021; J. Kim & Ortiz, 2024; Kuss et al., 2022; McLean & Griffiths, 2019; Moura et al., 2024). This support, whether through friendships within the game itself, specific communities or trusted environments outside the digital realm, not only alleviates the impact of violence, but also reinforces the decision to remain in the gamer space without renouncing one’s own identity.

However, women’s perception is that there is a lack of both instrumental and emotional social support from other players. These results can be observed in a study by McLean and Griffiths (2019). Women, rather than being part of collaborative communities, must protect themselves from sexist comments, mockery or disqualifications: “Better to play alone than subject myself to potential toxicity” (post 185, Woman, p. 978).

Along the same lines, Kuss et al. (2022) addresses social support, but in a positive way. Participants note that gaming has allowed them to establish and strengthen positive social bonds, which have had beneficial effects on their emotional and mental wellbeing:

It brings people together because you have a shared interest, it’s a good way to joke together, it’s good stress relief, and honestly, for a lot of people who don’t have much or who just aren’t happy in life, video games can offer a great escape

(FG6, Woman, p. 8).

This social support intensifies in the search for communities of female players who intentionally seek to create spaces that are free from discrimination and emotionally safe for everyone: “I would like to explore the world of digital video games, maybe find some multiplayer communities where I feel comfortable…” (FG7, Woman, p. 12). This search reflects a need for participation and belonging in discrimination-free spaces.

### 3.3. Question 3: Ambivalence and Internal Tensions

As a consequence of violence in gaming spaces, women’s journeys are marked by internal tensions that shape their identities and experiences in these communities. Their ambivalence is addressed in 67% (*n* = 10) of the analyzed studies, and manifests both on an individual and collective level (Choe et al., 2020; Deng, 2024; Giolla, 2018; S. J. Kim & Kim, 2022; Kuss et al., 2022; McLean & Griffiths, 2019; Moura et al., 2024; Naidoo et al., 2020; Ochsner, 2017; Ortiz, 2018). This reveals how female players constantly struggle between the desire to belong and the need to protect themselves, between passion for video games and the masculine norms that dominate the environment.

One of the most frequent forms of ambivalence is the difficulty in identifying whether certain situations are really sexist or whether women are being “too sensitive.” Women tend to evaluate their aggressor’s intentions before responding, which reveals emotional management conditioned by doubt and what others will say. In Deng’s (2024) study, women constantly question whether what they have experienced is really a discriminatory situation or is something “normal” in the community: “Somehow I perceive it, but I can’t report it for sexual discrimination, because men use those words constantly. I don’t want to be arbitrary, am I being too sensitive?” (Soupe, Woman, p. 10).

In the professional realm, this ambivalence is more acute. Women who work in the video game industry feel that their physical appearance can become a double-edged sword: the more attractive they are, the less competent they are considered: “My work performance is evaluated according to how attractive I am. The more attractive they consider me, the less competent I must be” (Woman, in Ochsner, 2017, p. 9). Some come to assume the role of “sexy girl” as a survival strategy to avoid reprisals, although this strategy reinforces gender stereotypes (Fousek & Švelch, 2024). These women live in a permanent state of “never being good enough,” a sense of insufficiency that affects both female players and professional women in the industry: “We have to play more, know more. And you know you’re never going to be good enough” (Jana, Woman, in Fousek & Švelch, 2024, p. 460). Some even suggest having adopted attitudes of differentiation toward other women, as a strategy to gain male acceptance: “I tend to do that sometimes, for example: ‘Guys, look at me, I’m great! Love me!’” (Jana, Woman, p. 459).

However, even within this will to resist, internal contradictions persist. Some female players consciously use behaviors associated with femininity or even sexualization to obtain benefits within the game. This ambivalence between reproducing and rejecting stereotypes does not respond to a lack of awareness or sensitivity, but to a logic of survival in an environment that is structurally hostile:

If we show our face or part of our body, they will give us gifts. For example, in LoL (League of Legends), they can give you skins, or in other types of games, it can be gold… if they want us to do something for them, it involves sexualization, you know?

(Woman, in Moura et al., 2024, p. 26).

The community also operates as a space of symbolic exclusion when what predominates is the discourse of “everyone receives abuse equally,” hiding the difference between general toxicity and sexism: “Many guys are verbally harassed just as much or more than women” (Anonymous, in Giolla, 2018, p. 128). This discourse minimizes gender violence by equating it with general toxicity, making its structural dimension invisible.

For this reason, many women just want to enjoy the game as a form of leisure, without being forced to turn their experience into a constant struggle or a topic of debate. This attitude does not imply a lack of awareness of the problem; it is simply a protective attitude in the face of the emotional exhaustion that resisting and fighting entails. This is captured in the study by Moura et al. (2024) under a topic called “girls just want to have fun” where women express:

Many times I play for fun, leisure and entertainment, so I don’t care about winning or losing. I play for myself, not for others, so I don’t do streams because I don’t care who is watching; what matters is who is playing with me in that match…

(Woman, p. 25).

Finally, tensions are also observed among men: some men express empathy toward harassed women, but do not act for fear of losing their status in the group: “No one will play with you if you stand up and say stop it! They’ll think you’re corny” (Frederick, in Ortiz, 2018, Man, p. 9).

## 4. Discussion

**Figure 2:**
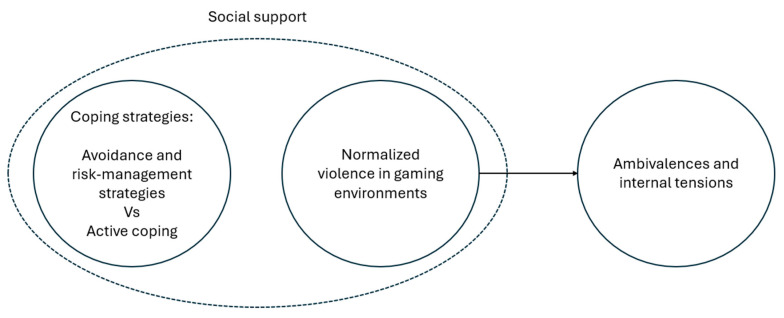
Interpretive model of principal results. Author’s elaboration based on the review of the selected studies.

This metasynthesis presents a global synthesis of published qualitative studies on sexism in video games in *gamer* communities. The results suggest that sexism is deeply rooted and normalized in video game culture and allow for a more subjective understanding of it. Sexist forms of aggression (along with others such as racism or homophobia) are specific forms of toxicity that many players justify them as something inherent to video games. Based on these findings, we constructed an interpretative model that illustrates the main dynamics that structure female players’ experiences (Figure 2). To understand sexism in video games, it is important to know the forms of violence that are normalized in these environments, the strategies female players use to confront this and the impact it has on them.

Based on the qualitative review of the fifteen empirical investigations included, this metasynthesis identifies recurring patterns, tensions, and ambivalences, as well as coping strategies that illuminate how sexism is enacted, perceived, and ultimately normalized within gaming environments. Importantly, these strategies can be interpreted not only as individual coping responses, but also as expressions of agency and everyday resistance shaped by gendered power relations. By consolidating fragmented qualitative findings, this metasynthesis addresses previously noted gaps in the literature and offers a more comprehensive and coherent map of how sexism affects women in gaming cultures and communities. In addition, the synthesis brings into view male discourse in video games and the ways violence and harassment become routinized, underscoring the importance of examining masculinities and how dominant masculine norms structure gaming spaces. Finally, the recurrent emphasis on informal networks of support highlights collective dimensions that are central to feminist approaches—namely, solidarity, community-building, and collective empowerment—alongside individual forms of empowerment (Kabeer, 1999). According to the Ambivalent Sexism Theory of Glick and Fiske (1996), sexism in video games manifests in two forms: hostile and benevolent. In hostile sexism, female players report high levels of verbal harassment, insults or sexist behaviors. The most frequent is gender *trash talking*, characterized by insults explicitly directed at females, such as “whore” or “go to the kitchen,” which seek to exclude women in a masculinized environment. In fact, theories about sexual harassment in organizations (Fitzgerald et al., 1997) can be extrapolated to the video game context, as it is a space where men make more noise, and their activity is strongly associated with hegemonic masculinity, thus increasing the probability that women will be victims of sexual harassment, another manifestation of hostile sexism in this realm.

As the results suggest, the lack of consequences for aggressors reinforces this normalization; video games are a safe space for men where hypermasculine behaviors, such as aggressive competitiveness or demonstrations of dominance, are the norm. Therefore, the female presence in competitive games is perceived by some men as a threat to their status. The results of this metasynthesis are in line with what previous studies affirm: men with lower skill tend to be more hostile toward women who surpass them, as they consider them an invasion of their competence and territory (Kasumovic & Kuznekoff, 2015). There are even female players who may come to accept these rules as something natural in *gamer* culture, unintentionally reinforcing the legitimacy of this masculinity. It could be said that there is a learned helplessness on the part of some women when they see that they cannot put an end to the problem, and therefore they accept it and consider it natural.

The results are in line with other systematic reviews where gender-based harassment in video games has been explored. For example, Yunhao et al. (2025), point out that masculinity or masculine traits predict the perpetration of cyberbullying, while femininity or feminine traits predict victimization. Although men can be victims of aggression in general, women have a higher probability of suffering harassment with a sexist component. It is important to note that these forms of violence do not affect all women equally. Some analyzed studies incorporate the intersectional perspective (Crenshaw, 1991), showing how gender intersects with other categories such as ethnicity or sexual orientation, intensifying the experiences of discrimination and violence. From this perspective, in online gaming communities voice plays a crucial role. Gray (2012) illustrates that, once a woman’s voice is heard, she often faces racialized sexism, a term reflecting the intersectional oppression where woman of color are identified by their voice and attacked for the duality of their identities. Indeed, some participants point out that when they confront or respond to the men who attack them, they are immediately labeled as lesbians. This represents a form of sexism where a woman who refuses to remain silent is stereotyped as not feminine enough. Therefore, she is perceived as masculine and categorized as a sexual minority as a way to undermine her presence.

These intersecting oppressions create a double or triple burden for women. They are not just fighting for a place in the game; they are navigating a virtual landscape that reproduces the systemic inequalities of the real world. This evidence highlights how safety in gaming is a privilege often reserved for those who fit the default profile, while others must actively labor creating their own communities and strategies simply to enjoy the same experience. However, this is not a struggle that only concerns women: intersectionality affects all people, as it defines how power and vulnerability are distributed in human interactions (Collins, 2017). Even those who fit the default profile are pressured to perform a specific narrow version of masculinity to remain safe within the group, as the study from this metasynthesis regarding men’s experiences analyze. This suggests that intersectionality is not merely a framework for understanding victimization, but a tool to reveal how rigid power structures restrict the authentic expression and human connection in gaming communities.

On the other hand, benevolent sexism is also present as a form of normalized violence, although it is seen as “positive.” The most common expression of this form of sexism is assuming that women should play support roles. This type of thinking reproduces the stereotype of women as caregivers and empathetic, traits that are positive but that limit female players’ performance and perpetuate the division of roles. Another thing that can be implicit in benevolent sexism is the help that men provide to female players without them requesting it. In turn, if a woman excels in the game men are surprised, as if they wouldn’t be expected to excel. This behavior, although apparently kind, reinforces the idea that women are not as capable as men. Finally, some players offer gifts to female players only for fulfilling a sexualized role, or they simply receive romantic or sexual insinuations. This sexist behavior treats women as if they were an object or a complement to men, although “it’s done with good intentions.” Furthermore, women’s refusal of these solicitations can lead to potential harassment. That is, not accepting benevolent sexism as something good can give rise to hostile sexism.

Other systematic reviews that have been conducted on women’s experiences in video game environments (García-Naveira et al., 2022; Gestos et al., 2018) also showed a clear sexualization of women: the male gaze perpetuates gender stereotypes associated with femininity. Research highlights that this sexualization has an impact on the real lives of female players, fostering ideas of misogyny or sexual abuse toward them. The discourses analyzed in this metasynthesis agree in pointing out a common reality: female players find themselves immersed in a hostile environment, characterized by sexualization, harassment and patriarchal mechanisms that seek to silence them and limit their participation.

In this hostile and gendered context, female players describe developing multiple coping strategies that enable them to remain in gaming spaces while managing the risk of harassment. From a feminist perspective, these practices can be understood as forms of agency and everyday resistance enacted under unequal power relations, rather than merely individual adaptations to an “aggressive” environment (Kabeer, 1999; Mahmood, 2005). Consistent with prior systematic reviews (García-Naveira et al., 2022; Vergel et al., 2023; Yunhao et al., 2025), the findings of this metasynthesis suggest that the most frequently reported avoidance strategy is gender masking or concealment. Many women adopt gender-neutral pseudonyms or masculine avatars and refrain from using voice chat to pre-empt insults, unwanted sexual advances, and other forms of sexist treatment. While such strategies may reduce exposure to harassment, they can also require women to limit visibility and self-expression, potentially undermining identity affirmation and belonging and generating the ambivalences and tensions reported across studies.

Relatedly, women frequently describe avoiding—or exiting—spaces perceived as toxic as a way of protecting themselves and sustaining participation over time. Some prefer to play online only with trusted groups, whereas others avoid competitive modes altogether; others shift to single-player games, thus removing exposure to hostile interactions. Importantly, these patterns point to structural costs of sexism in gaming cultures: in competitive settings, persistent hostility is associated with progressive disengagement, which may help explain women’s underrepresentation in arenas such as eSports. At the same time, some women report more confrontational strategies, including calling out aggressors or adopting a tougher, more aggressive stance. Although these responses may provide short-term protection or credibility, they can also entail aligning with dominant masculine norms and performative toughness (Butler, 1990; Connell, 1995; Connell & Messerschmidt, 2005), thereby illustrating a central feminist tension between survival within hostile environments and the potential reproduction of the very norms that sustain them. Relatedly, accounts across studies highlight the ongoing requirement to demonstrate competence and legitimacy, adding a gendered burden of proof and emotional labor that exceeds what is expected of male players.

Beyond these individual practices of risk management and identity negotiation, the metasynthesis highlights the collective dimensions that feminist scholarship identifies as central to empowerment: solidarity networks, community-building, and mutual support. Across the included studies, women’s efforts to create safer and more inclusive spaces can be read as forms of collective resistance that counter isolation and expand both individual and collective empowerment. Evidence from the gender-based violence literature indicates that social support is associated with a lower likelihood of victimization (Huang et al., 2010), and similar protective dynamics have been discussed in gaming contexts (García-Naveira et al., 2022; Vergel et al., 2023). As participants suggest, building supportive communities is therefore a key community-level strategy that may buffer the impacts of harassment and other forms of gendered violence, enabling continued participation rather than withdrawal. Nevertheless, feminist analyses also remind us that access to such networks is unequally distributed: building and sustaining solidarity can be difficult in male-dominated environments. Initiatives such as #PlayEquall point to ongoing progress in this direction, while also underscoring the need for structural changes that do not place the burden of safety solely on women. Consequently, ambivalences and internal tensions emerge that affect the wellbeing of female players. In line with Ambivalent Sexism Theory, many constantly evaluate whether certain behaviors are sexist, especially when it comes to benevolent sexism, which generates doubts, social tolerance and, in some cases, silence as a strategy to avoid conflicts. Additionally, female players experience internal tensions linked to their identity and the mixed messages they receive. If women play poorly, they are attacked with the fact that they don’t belong to the community, but if they play well, they are attacked for showing more skill than men. The results suggest that this clash translates into ambivalent feelings. Some report whether it’s really worth staying where they are not welcome, while giving up would imply abandoning an identity where video games are their passion. This also manifests in the industry, as this analysis suggests. In this sense, some female players and professionals end up expressing qualities that they don’t want to see linked to the “female *gamer*.” This analysis suggests that there are female players who opt to adopt stereotypes traditionally linked to femininity in terms of their identity. This is also supported by recent studies along the same lines, both in the reproduction and acceptance of “female *gamer*” stereotypes (Yao & Rhodes, 2023) and self-sexualization (Anciones-Anguita & Checa-Romero, 2024). In a certain way, there exists a “patriarchy by resignation” where women, tired of resisting, choose to accept and adapt to the hegemony.

Unlike systematic and/or integrative reviews on this topic (García-Naveira et al., 2022; Moreno-López & Argüello-Gutiérrez, 2025; Vergel et al., 2023; Yunhao et al., 2025), this metasynthesis addresses sexism in the realm of video games from an innovative perspective that delves into the lived experiences and subjective interpretations of players, both victims and witnesses. The results illustrate symbolic and relational nuances, which highlights the ambivalences and internal tensions that appear in female players. The metasynthesis methodology provides a complementary value to other types of review, which is an approach to the life experiences, perceptions and coping strategies of the players themselves.

### 4.1. Limitations

In terms of the limitations of the present work, there are few qualitative studies that address the topic of sexism in online video games. As the first metasynthesis conducted on this topic, it is difficult to establish prior comparisons or validate the current findings with prior studies of similar nature. Likewise, a large part of the studies come from specific (Anglo-Saxon) sociocultural contexts, which may limit transferability of the results. On the other hand, a lack of intersectional perspective in some of the articles may limit an understanding of how sexism and violence operate intersectionally, making future qualitative work that applies this perspective necessary. Furthermore, there is a lack of research on this topic with the male population, so understanding the male vision of the problem is complex within a single investigation. Finally, while this metasynthesis incorporates the voices of industry stakeholders such as game designers, journalists, and eSports professionals, it does not specifically address player perceptions of institutional anti-harassment protocols. Given the notable gap in the literature regarding how users interpret these measures, these findings should be considered a foundational step for future research to analyze the effectiveness and reception of such corporate policies.

### 4.2. Practical Implications and Possible Lines for Future Research

The findings presented in this study offer practical and social implications. First, they highlight the urgent need to prevent and combat sexism in *gaming* spaces, using a comprehensive perspective and a multidimensional approach (Becker et al., 2014). At the practical level, the findings can inform the design of regulations and protocols in *gaming* platforms (such as Blizzard, Activision, Microsoft, etc.) and player communities from a more subjective point of view of the experiences of female players, male players and the industry. Currently, online video game companies have reporting and account suspension systems in place, although it appears they are not effective enough to end the structural problem and toxicity in general. On the other hand, the analysis conducted in this work can be used for internal training in the video game industry, creating good practice guidelines that promote more inclusive and egalitarian environments.

Another important issue concerns the education of young people. This study can be a useful resource to inform people about the problem of sexism in video games, so that educational programs can be created to address digital violence. It can also be useful to train teachers, counselors and families about the exposure of children and adolescents to violent situations in video games. Additionally, it can also serve as a resource for developing individual coping skills or supportive resources for communities of female players, thus promoting the empowerment of women in the video game sector.

At the social level, it is hoped that this work will make visible and name the forms of violence that women experience in the video game context, contributing to their eradication. The work provides arguments for the transformation of the *gamer* community into a community where equality, respect, diversity and inclusion of all people are valued. Additionally, this analysis represents an important advance for research on gender and technology, as it addresses current qualitative research on sexism and video games. Furthermore, this metasynthesis could be used for inclusion in qualitative or quantitative studies in this field.

In terms of future research, these results highlight various future research lines that could address a gap in information. For example, the psychosocial effects of sexism in online video games have not been studied qualitatively, nor have anxiety, women’s self-efficacy, etc., been sufficiently addressed.

Another area of research where there is practically no information is the study of male perspectives. That is, there is little research concerning the role of men in digital sexism or the diverse masculinities that can surround video games. Finally, the grouping of qualitative studies presented here can be of interest, along with quantitative studies, for the creation of evaluation instruments or scales on sexism in this field. Furthermore, although not specifically addressed, it can also support research on gender representations in video games and how they affect people’s perceptions.

## Figures and Tables

**Table 1:** Number of articles selected for the metasynthesis (*n* = 15).

Authors	Country	Year	Sample	Objective	Collection Method
Cote, A. C.	USA	2015	37 women	To explore the coping strategies of women who experience sexist harassment in online video games	Semi-structured interviews
Crothers, H., Scott-Brown, K., & Cunningham, S.	UK	2024	8 women	To explore how experiences of sexism and toxicity affect women who participate in eSports	Semi-structured interviews
Deng, Z.	UK	2024	40 women	To explore how female video game players understand sexism, how they react to it and what their coping strategies are	Semi-structured interviews
Fousek, T., & Svelch, J.	Czech Republic	2024	8 women	To analyze how female journalists view their position in video game culture and what strategies they use to confront sexism	Semi-structured interviews
Harvey, A.	Canada	2021	14 women students and 4 women teachers	To investigate how women in higher education video game programs confront masculinized spaces and cultures	Semi-structured interviews
Kim, J., & Ortiz, N.	USA	2024	19 men, 3 women and 1 non-binary person	To examine how video game players confront and resist toxic behaviors in community contexts	Semi-structured interviews
Ochsner, A.	USA	2019	1920 comments on the platform “X” (formerly Twitter)	To examine the #1ReasonWhy conversation to discover how video game industry professionals and people in video game communities explain why so few women choose to pursue or remain in careers in the video game field	Observation of comments and descriptive coding of the text
Ortiz, S.	USA	2018	12 men	To analyze the meaning of trash talk with racist and sexist content that men exhibit within the gamer community	Semi-structured interviews
Choe, K., Doh, S. J., & Ha, J.	South Korea	2020	15 women and 10 men	To explore the experiences of sexism in online games among adolescents and to understand how it affects them	Semi-structured interviews, focus groups and online interviews via mobile phone
Giolla, B. N.	Australia	2018	Multimodal data collected from texts (26), podcasts (2) and videos (3)	To explore how sexism is understood and negotiated in online gaming communities, and to analyze the perceptions and disputes around gender-based harassment and player identity	Network analysis software (IssueCrawler), Observation of textual data complemented with grounded theory for analysis
Naidoo, R., Coleman, K., & Guyo, C.	South Africa	2020	200 comments in 8 discussion threads	To analyze the discursive gender struggles over social inclusion in an online gaming community, identifying dominant discourses and marginalized discourses	Observation of messages with contrapuntal analysis technique
McLean, L., & Griffiths, M.	Ireland	2019	271 women players, via 1043 publications in forums	To explore the experiences of online harassment and social support of female players, identifying coping strategies and the impact on their psychological wellbeing	Observation in forums with thematic analysis
Kim, J., & Kim, S.	USA	2022	11 women and 9 men	To explore the social barriers that limit women’s participation in eSports and propose strategies to promote their inclusion	Semi-structured interviews and open-ended surveys
Kuss, D., Kristensen, A. M., Williams, J., & Lopez-Fernandez, O.	UK	2022	20 women	To explore in depth the experiences of women who play video games regularly, addressing the construction of their identity as players, the benefits and risks associated with gaming, and their future perspectives	Semi-structured interviews
Moura, B. M., Souza-Leão, A. L., M. de., Salgueiro, E. M. G., Crosato, M. S., & Rocha, A. L. da S.	Brazil	2024	44 women	To investigate how gender performativities are produced in the consumption of eSports by female gamers	Semi-structured interviews

**Table 2:** Reciprocal translation process of the identified studies (*n* = 15).

Author	Preliminary Theme	Key Theme: First Interaction	Final Theme: Second Interaction
Cote (2015)	– Abandonment – Avoidance of strangers – *Gender masking* – Emphasis on skill – Adoption of aggressive roles	– Passive avoidance responses – Active confrontation responses	– Strategies for coping with sexism/gender-based harassment
			
Crothers et al. (2024)	– Experiences of exclusion – Stereotypes roles – Systemic gender-based harassment – Gender masking – Community and inclusion	– Reproduction of stereotypes – Normalization of violence toward women – Active confrontation responses	– Normalized violence in gamer environments – Strategies for coping with sexism/gender-based harassment
			
Deng (2024)	– Hypersexualization – Sexual harassment outside the game – Mysogeny in gaming forums – Discrediting women’s skills – Gender masking – Abandonment – Lack of recognition of sexism	– Reproduction of stereotypes – Normalization of violence toward women – Passive avoidance responses – Legitimation of violence	– Normalized violence in gamer environments – Strategies for coping with sexism/gender-based harassment – Ambivalence and internal tensions
			
Fousek and Švelch (2024)	– Gender masking – Fetishistic role of women – Fake “geek girl” – Different treatment – Ignore, fight or distance oneself	– Passive avoidance responses – Active confrontation responses – Reproduction of stereotypes – Normalization of violence toward women	– Strategies for coping with sexism/gender-based harassment – Normalized violence in gamer environments
			
Harvey (2021)	– Exceptionality – Hostility – Alignment with gamer identity – Attempt to fit in – Refusal to maintain the problem – Social support and solidarity networks and women’s initiatives	– Normalization of violence toward women – Active confrontation responses – Importance of social support	– Normalized violence in gamer environments – Strategies for coping with sexism/gender-based harassment
			
J. Kim and Ortiz (2024)	– Tolerable toxicity – Intolerable toxicity – Confrontation strategies – Importance of community	– Gradation of harassment – Active confrontation responses – Importance of social support	– Normalized violence in gamer environments – Strategies for coping with sexism/gender-based harassment
			
Ochsner (2017)	– Different evaluations of women – Double standards – Harassment in the university – Harassment at business events and conferences	– Differential gender norms – Institutional sexual harassment – Violence in professional environments	– Ambivalence and internal tensions – Normalized violence in gamer environments
			
Ortiz (2018)	– Trash talk as gaming capital – Trash talk as cultural practice – Sexism as domination within the game – Invisibilization of sexism that men experience	– Denial of sexism – Normalized symbolic harassment – Differential gender norms	– Ambivalence and internal tensions – Normalized violence in gamer environments
			
Choe et al. (2020)	– Gender stereotypes in skills – Exclusion of women – Confrontation or resignation – Masculinization of the environment – Adapted behaviors – Desire for inclusion vs. fear of rejection	– Normalization of violence toward women – Active confrontation responses – Passive avoidance responses – Differential gender norms	– Ambivalence and internal tensions – Strategies for coping with sexism/gender-based harassment – Normalized violence in gamer environments
			
Giolla (2018)	– Unequal recognition of sexism – Symbolic violence – Gender as a category – Obscured intersectionality – Privileges – Differentiated criteria	– Differential gender norms – Normalized symbolic harassment – Denial of sexism	– Normalized violence in gamer environmentsAmbivalence and internal tensions
			
Naidoo et al. (2020)	– Exclusion due to harassment – Exclusion due to character stereotypes – Exclusion due to masculine hypercompetitiveness – Exclusion in industry employment networks	– Normalized symbolic harassment – Denial of sexism – Differential gender norms – Reproduction of stereotypes	– Ambivalence and internal tensions – Normalized violence in gamer environments
			
McLean and Griffiths (2019)	– Lack of social support – Gender masking – Avoidance – Psychological impact – Putting skills in question – Internal and external pressure	– Importance of social support – Psychological consequences – Passive avoidance responses – Normalized symbolic harassment	– Strategies for coping with sexism/gender-based harassment – Ambivalence and internal tensions
			
S. J. Kim and Kim (2022)	– Entry trajectory into gamer culture – Gender stereotypes – Gender-based harassment – Differential treatment – Motivational differences – Tension with gamer identity – Importance of social support and solidarity networks and female visibility	– Reproduction of stereotypes – Differential gender norms – Importance of social support – Normalization of violence toward women	– Normalized violence in gamer environments – Ambivalence and internal tensions
			
Kuss et al. (2022)	– Questioning of female gamer identity – Gender stereotypes – Gender masking – Avoidance – Pride and self-acknowledgement – Positive impact of gaming – Gaming risks – Women’s visibility	– Reproduction of stereotypes – Passive avoidance responses – Active confrontation responses – Importance of social support	– Ambivalence and internal tensions – Strategies for coping with sexism/gender-based harassment
			
Moura et al. (2024)	– Discrimination and repeated harassment – Invisibilization of sexism – Struggle for spaces of equality – Confrontation vs. silence – Social support and solidarity networks – Prioritization of entertainment over performance – Reproduction of gender roles	– Reproduction of stereotypes – Passive avoidance responses – Importance of social support – Normalized symbolic harassment	– Normalized violence in gamer environments – Strategies for coping with sexism/gender-based harassment – Ambivalence and internal tensions

**Table 3:** Evaluation of study quality according to the Mixed Methods Appraisal Tool (MMAT; Hong et al., 2018).

Criteria	Yes	No	I Don’t Know
Are there clear research questions/objectives?	15	0	0
Does the data collected allow for answering the study questions?	15	0	0
Is the qualitative approach adequate for answering the research question?	15	0	0
Are the data collection methods adequate for addressing the research question?	15	0	0
Are the findings adequately derived from the data?	15	0	0
Is the interpretation sufficiently supported by the data?	15	0	0
Is there coherence between sources, collection, analysis and interpretation?	15	0	0

## Data Availability

Data sharing is not applicable.
